# First Isolation of Bovine Coronavirus From Yanbian, China, and Analytical Validation of a SYBR Green I RT‐qPCR Panel for Calf Diarrhea Viruses

**DOI:** 10.1155/tbed/6648536

**Published:** 2026-04-27

**Authors:** Siqi Zhang, Haoyuan Ma, Jiawei Zhao, Kai Yu, Jingrui Hao, Hao Yu, Jianyou Jin, Shuoning Cao, Xinpeng Ji, Shujiang Xue, Qiang Li, Zhiqiang Xu, Shengwei Ji, Chenghui Li, Zheng Sun, Jialiang Xie, Rui Du, Xu Gao

**Affiliations:** ^1^ Laboratory for Animal Molecular Virology, Department of Veterinary Medicine, College of Agricultural, Yanbian University, Yanji, 1330002, China, ybu.edu.cn

**Keywords:** bovine coronavirus, differential diagnosis, neonatal calf diarrhea, regional surveillance, SYBR Green I RT-qPCR

## Abstract

Bovine coronavirus (BCoV) is an important viral agent associated with neonatal calf diarrhea (NCD), winter dysentery, and bovine respiratory disease, contributing to economic losses in cattle production worldwide. To date, BCoV sequence data from Yanbian, Northeast China, are limited in public datasets. In this study, we report the first successful isolation of a BCoV strain (BCoV‐YBYJ) from a diarrheic calf in Yanbian using MDBK cells and characterize its replication in vitro and describe exploratory colon and lung findings following high‐dose oral exposure in a murine model. The isolate replicated efficiently in MDBK cells. In mice, viral RNA was detected in the colon and lung with mild‐to‐moderate histological changes under high‐dose exposure (nonnatural host). Phylogenetic analysis based on partial ORF1a, S, and M sequences showed that BCoV‐YBYJ clustered with contemporary Chinese isolates within the available reference set, supporting regional genetic relatedness based on partial loci. In parallel, three SYBR Green I‐based RT‐qPCR assays targeting BCoV, bovine rotavirus (BRV), and bovine parvovirus (BPV) were established and analytically validated. These assays demonstrated satisfactory analytical sensitivity, specificity, and reproducibility within the validated dynamic range, and BCoV RNA was detected in 70% (140/200) of targeted diarrheic calf specimens (*n* = 200). This study provides a Yanbian BCoV isolate and analytically validated RT‐qPCR assays to support differential detection and surveillance of major viral agents associated with calf diarrhea. Because these were targeted submissions from diarrheic calves, this proportion is reported descriptively and should not be interpreted as population prevalence. The BRV and BPV assays underwent analytical validation only and require further clinical evaluation before routine diagnostic application.

## 1. Introduction

Neonatal calf diarrhea (NCD) is one of the most prevalent and economically important diseases affecting cattle production worldwide [1]. It is widely recognized as a multifactorial condition in which multiple enteric pathogens may be involved, including bovine rotavirus (BRV), bovine parvovirus (BPV), and bovine coronavirus (BCoV), frequently in combination with bacterial (e.g., enterotoxigenic *Escherichia coli*) and parasitic agents (e.g., *Cryptosporidium* spp.) under field conditions. The relative contribution of individual pathogens, however, varies across herds, geographic regions, and outbreak settings.

Among NCD‐associated viruses, BCoV is distinctive in exhibiting a dual enteric and respiratory tropism. In addition to its role in calf diarrhea, BCoV has been implicated in winter dysentery in adult cattle and in bovine respiratory disease complexes across age groups. Infected animals may shed the virus via both fecal and respiratory routes, facilitating within‐herd dissemination and contributing to the persistence of BCoV in cattle populations.

The successful isolation of BCoV strains remains essential for generating reference materials for diagnostic assay development, monitoring genetic and antigenic variation, supporting vaccine evaluation, and advancing the understanding of viral biology. Previous studies have reported the recovery of respiratory and enteric BCoV strains from clinically affected cattle [2, 3], highlighting the continued circulation of genetically diverse lineages, even in vaccinated herds. However, data from border regions characterized by frequent livestock movement remain limited, underscoring the need for localized molecular surveillance.

Accurate laboratory diagnosis of BCoV in field samples remains challenging. Coinfections with BRV and BPV can result in overlapping clinical manifestations, complicating etiological attribution [4]. Traditional diagnostic approaches such as virus isolation and antigen‐based assays may exhibit limited sensitivity under routine conditions, particularly when viral loads are low. While conventional PCR is widely used, it can be less sensitive than RT‐qPCR under routine conditions, particularly when viral loads are low or inhibitors are present in field specimens. In this context, SYBR Green I‐based real‐time RT‐qPCR offers a sensitive and rapid molecular alternative, with melt‐curve analysis enabling verification of amplicon specificity in single‐target assays [5, 6].

In the present study, we report the first successful isolation of a BCoV strain (BCoV‐YBYJ) from a diarrheic calf in Yanbian, a region located at the Sino–Korean–Russian border. We further characterized its replication kinetics in cell culture and descriptively examined colon and lung findings following high‐dose oral exposure in a murine model as an exploratory in vivo system. In parallel, we established and analytically validated three SYBR Green I‐based RT‐qPCR assays targeting conserved regions of BCoV (ORF1a), BRV (VP6), and BPV (NS1) [7] using clinical samples. Together, these data provide a locally derived isolate, partial‐locus phylogenetic context, and analytically validated assays to support research surveillance and differential screening of major viral agents associated with NCD [8, 9].

## 2. Materials and Methods

### 2.1. Cell Culture

MDBK cells (Zhejiang Beidibio, China) were authenticated for species origin and confirmed to be mycoplasma‐negative by PCR (tested monthly). The cells were maintained at 37°C in a humidified incubator with 5% CO_2_ in DMEM (Baidi, China) supplemented with 10% heat‐inactivated fetal bovine serum (56°C, 30 min; Baidi, China) and 1% penicillin‐streptomycin. MDBK cells were used between passages P10–P25. Unless otherwise stated, monolayers at 80%–90% confluence were used for infection.

### 2.2. Sample Collection and Processing

Nasal and anorectal swabs were collected from diarrheic calves at multiple cattle farms in Yanji, Jilin Province, China, to capture potential respiratory and enteric shedding routes [10]. The sampling protocol was reviewed and approved by the Institutional Animal Care and Use Committee (IACUC) of Yanbian University (Approval Number YD20250827010). The 200 field specimens analyzed by RT‐qPCR were obtained from diarrheic animals during routine veterinary investigations and were used here to describe Ct distributions rather than to establish diagnostic performance. Swabs were washed with PBS, diluted 1:10 in PBS, homogenized, clarified at 4000 × *g* for 30 min at 4°C, and the supernatants were passed through 0.22 µm filters (Millipore) to reduce bacterial contamination prior to downstream processing [11] and stored at −80°C.

### 2.3. Clinical Swab RNA Extraction, Reverse Transcription, and BCoV‐Specific PCR Confirmation

Total RNA was extracted from clinical swab samples using TRIzol reagent (Thermo Fisher Scientific, USA). cDNA was synthesized with PrimeScript RT Master Mix (Takara, Japan) following the manufacturer’s protocol. For sequence confirmation of the screening amplicon, BCoV‐specific PCR was performed using PrimeSTAR HS DNA polymerase (Takara, Japan), which was selected to minimize polymerase‐introduced errors prior to Sanger sequencing (this was not intended as a routine diagnostic PCR workflow) [12]. Reactions (25 µL) contained 3 µL cDNA, 12.5 µL 2× reaction mixture, 1 µL of each primer (10 µM; primer sequences are listed in Table 1), and nuclease‐free water to volume. Thermal cycling was 95°C for 5 min; 35 cycles of 95°C for 30 s, 55°C for 30 s, and 72°C for 15 s; and a final extension at 72°C for 10 min. PCR products were resolved on 1% agarose gels; samples yielding a single band of expected size were gel‐purified and confirmed by Sanger sequencing. Only sequencing‐confirmed BCoV‐positive samples were used for subsequent virus isolation.

**Table 1:** The primers used in this study.

Primers	Primer sequences (5′–3′)	Product size (bp)
BCoV‐ORF1a	F: AGGAACACCTATTGCCAATTG	216
	R: GGATCTTTTATACCTACAGGCAC	
BPV	F: CCAATCGTCCTCTACTGCTT	110
	R: GTGCTCGGTGAGCGCTAAAT	
BRV	F: AGAAGACAAAGAACGGGTTT	113
	R: CACATCGTACCCATCAAGTTAT	
β‐Actin (bovine)	F: GATGATATTGCTGCGCTCGTG	131
	R: CCCACCATTACGCCCTGG	
GAPDH (*Mus musculus*)	Commercial primer set Beyotime, China; Cat. No. OM00014S	Not disclosed

### 2.4. Virus Isolation in MDBK Cells

Clarified material from PCR‐confirmed positive samples was inoculated (5% v/v inoculum) onto MDBK cell monolayers at ~ 90% confluence and incubated at 37°C in a humidified 5% CO_2_ atmosphere, as commonly used for BCoV isolation and characterization [13]. Mock‐infected cells served as negative controls. Supernatants from cultures showing cytopathic effects (CPEs) were clarified and passaged into fresh MDBK cells for up to 10 passages. At each passage, 200 µL of culture supernatant was collected. Virus‐positive stocks were aliquoted and stored at −80°C.

### 2.5. Virus Titration and Growth Kinetics

The 10th‐passage BCoV‐YBYJ stock was serially diluted tenfold (10^−1^–10^−9^) in serum‐free DMEM and inoculated (100 µL/well) onto confluent MDBK monolayers (2 × 10^4^ cells/well, preseeded 24 h earlier) in 96‐well plates, with eight replicate wells per dilution. After 2 h adsorption at 37°C, inocula were replaced with maintenance medium (DMEM + 1% FBS), and cultures were incubated at 37°C with 5% CO_2_. Wells were examined daily for up to 120 h postinfection (h.p.i.) for CPE, and infectious titers (TCID_50_/mL) were calculated by the Reed–Muench method based on CPE‐positive/negative scoring.

For single‐step growth kinetics, MDBK cells were infected with BCoV‐YBYJ at a multiplicity of infection (MOI) of 5. Culture supernatants were harvested at the indicated time points from 0 to 96 h.p.i., infectious titers were determined as described above, and growth curves were plotted as the means ± SDs from three independent experiments.

### 2.6. Transmission Electron Microscopy (TEM)

BCoV particles (10th passage, 2 mL) were concentrated by ultrafiltration (100‐kD cutoff membrane). The concentrates were adsorbed onto copper grids, fixed with 2.5% glutaraldehyde, negatively stained with 2% phosphotungstic acid, and examined using a Hitachi HT7700 transmission electron microscope at 180 kV. At least 100 particles were measured across three replicates.

### 2.7. Immunofluorescence Assay (IFA)

MDBK cells grown on coverslips were infected with the 10th‐passage BCoV‐YBYJ stock at an MOI of 1. At 72 h.p.i., the cells were fixed with 4% paraformaldehyde, permeabilized with 0.1% Triton X‐100, and blocked with 5% BSA. The cells were incubated with a mouse anti‐BCoV polyclonal antibody (in‐house preparation, dilution 1:500), followed by incubation with TRITC‐conjugated goat anti‐mouse IgG (Affinity, China, 1:200). The fluorescence signals were visualized with a fluorescence microscope (Olympus, Japan). Mock‐infected MDBK cells served as negative controls.

### 2.8. Phylogenetic Analysis

The ORF1a fragment amplified for RT‐qPCR (221 bp) was also subjected to bidirectional Sanger sequencing for sequence confirmation and phylogenetic analysis. After trimming primer sequences and low‐quality ends, a 216‐bp high‐quality consensus ORF1a fragment was obtained (GenBank: PX259842.1). In addition, fragments of the S (designed amplicon 1316 bp) and M (designed amplicon 693 bp) genes were amplified by RT‐PCR and Sanger‐sequenced bidirectionally. After trimming primer sequences and low‐quality ends, consensus sequences of 1320 bp (S) and 672 bp (M) were obtained for phylogenetic analysis and GenBank submission (Accession Numbers PZ099269 [S] and PZ099268 [M]). Partial nucleotide sequences were aligned with representative GenBank references in MEGA X, and maximum‐likelihood (ML) trees were inferred for ORF1a (216 bp), S (1320 bp), and M (672 bp) fragments with 1000 bootstrap replicates to assess branch support. Primer sequences used for amplification and Sanger sequencing of the partial S and M gene fragments are provided in Supporting Information 2: Table S2.

### 2.9. BALB/c Mice Infection Model

All animal procedures were approved by the institutional committee (IACUC Number YD20250827010). Male BALB/c mice (4 weeks; *n* = 3/group/time point) were housed in individually ventilated cages with ad libitum access to food and water. After 6 h of fasting, mice were orally inoculated with 4 × 10^8^ TCID_50_ of P10 BCoV‐YBYJ in 100 µL DMEM; mock controls received DMEM. Animals were allocated to infected or mock groups, and histopathological scoring was performed by blinded observers. At 1, 4, and 7 days postinfection (d.p.i.), mice were euthanized and tissues were harvested. For molecular quantification in the colon and lung, BCoV RNA was measured by RT‐qPCR and normalized to GAPDH; results are reported as log_10_(2^−ΔCt^), where ΔCt = Ct(BCoV) − Ct(GAPDH). For histopathology, tissues were fixed in 10% neutral buffered formalin and H&E stained. A blinded semiquantitative scoring system (0–4) was applied by five independent observers according to predefined criteria (tissue architecture, inflammatory infiltrates, and edema; see Supporting Information 3: Methods S1), and observer‐level scores are reported to visualize interobserver agreement. Given the limited biological sample size (*n* = 3 mice/time point), histopathology outcomes are presented descriptively. Immunohistochemistry (IHC) was performed on intestinal sections to assess viral antigen, and virus recovery was attempted from colon and lung tissues collected at 4 and 7 d.p.i. Tissues were homogenized in sterile PBS containing antibiotics, clarified by centrifugation, and the supernatants were passed through a 0.22 µm filter before inoculation onto confluent MDBK monolayers. In parallel, mock tissue homogenates from control mice processed using the same workflow were inoculated onto MDBK cells (mock‐MDBK control). Cultures were monitored for CPE, and culture supernatants were tested by RT‐qPCR for confirmation. These recovery attempts were intended as supportive evidence of recoverable viral material from tissues and were not designed to definitively establish in vivo replication. Data are presented as individual values with mean ± SD. Given the limited biological sample size in the mouse experiment, analyses are primarily descriptive; where exploratory comparisons were performed, appropriate parametric or nonparametric tests were used as applicable.

### 2.10. Plasmid Standard Generation

PCR amplicons of BCoV ORF1a, BRV, and BPV were cloned into pMD19‐T vectors (Takara) and confirmed by Sanger sequencing. Plasmid copy numbers were calculated using copies/µL = (*C* × 6.022 × 10^23^) / (*N* × 660), where *C* is plasmid concentration (g/µL) and *N* is plasmid length (bp) [14]. Ten‐fold dilution series (10^7^–10^1^ copies/µL) were prepared for standard curves and analytical validation [15].

### 2.11. Optimization and Analytical Validation of RT‐qPCR Assays

Conventional PCR assays for BCoV, BRV, and BPV were optimized (primer sequences in Table 1; in silico primer validation summarized in Supporting Information 1: Table S1) by testing primer concentrations (0.1–0.6 µM) and annealing temperatures (55–65°C). Detection limits were determined using 10‐fold serial plasmid dilutions. SYBR Green I RT‐qPCR assays were optimized by varying primer concentrations (0.2–1.0 µM). Standard curves (10^7^–10^1^ copies/µL) were used to evaluate linearity and amplification efficiency. A specimen was considered positive when it showed a typical sigmoidal amplification curve together with a single, specific melt‐curve peak at the expected *T*_m_. To minimize reporting of sporadic late‐cycle signals, we used a conservative primary cut‐off (Ct ≤ 35); signals with Ct > 35 were treated as borderline and were retested, and were reported as positive only if reproducible and melt‐curve consistent; otherwise, they were reported as negative/indeterminate. Reproducibility was assessed by intra‐ and inter‐assay CV across different days and operators. Interlaboratory repeatability was explored by testing the same plasmid dilution series in an independent laboratory using a harmonized wet‐lab protocol; because Ct calling (baseline/threshold) is instrument‐ and software‐dependent, interlaboratory results are interpreted primarily in terms of linearity/Ct concordance and not as definitive efficiency estimates (see Supporting Information 4: Results S3; raw interlaboratory standard‐curve data in Supporting Information 5: Data S3) in line with established real‐time PCR assay development practices [16] and prior calf diarrhea pathogen panel designs [17].

Field sample screening (descriptive). Nasal and anorectal swabs from diarrheic cattle were tested with optimized RT‐qPCR assays. Ct distributions and positivity (Ct ≤ 35 with the expected melt peak) were summarized descriptively. Conventional PCR was used as a qualitative comparator rather than for diagnostic performance estimation.

## 3. Results

### 3.1. Successful Isolation and Replicative Dynamics of BCoV‐YBYJ in MDBK Cells

PCR amplification using BCoV‐specific primers targeting the ORF1a yielded a distinct 216 bp band, confirming the presence of BCoV in the sequencing‐confirmed sample (Figure 1a). A PCR‐positive sample was subsequently selected for virus isolation.

Figure 1Isolation and in vitro characterization of the BCoV‐YBYJ strain. (a) RT‐PCR detection of ORF1a (216 bp). Lane M, DL2000 marker; lanes 1/2/4/5/7/8, positive samples; lanes 3/6, negative controls. (b) Cytopathic effects (CPE) in MDBK cells at 72 h.p.i. (right) compared with mock‐infected cells (left), showing cell rounding, shrinkage, and detachment from the culture surface (scale bars = 100 µm). (c) Single‐step growth kinetics of BCoV‐YBYJ in MDBK cells. Viral titers are expressed as log_10_ TCID_50_/mL (Reed–Muench). Peak titers occurred at ~24 h.p.i. Comparisons were performed against 0 h using one‐way ANOVA with multiple‐comparisons correction. Significance is indicated in the figure; ns, not significant;  ^∗^ *p* < 0.05;  ^∗∗^ *p* < 0.01;  ^∗∗∗^ *p* < 0.001;  ^∗∗∗∗^ *p* < 0.0001. (d) Time‐course of relative BCoV ORF1a RNA levels in MDBK cells quantified by RT‐qPCR (2^−ΔΔCt^, normalized to β‐actin and calibrated to mock‐infected cells; mean ± SD, *n* = 3). Statistics: one‐way ANOVA with Dunnett’s multiple‐comparisons versus mock control;  ^∗∗∗∗^ *p* < 0.0001. (e) Transmission electron microscopy image showing enveloped, pleomorphic coronavirus‐like particles bearing club‐shaped surface projections (spikes). Particle diameter ranged from 62 to 210 nm (mean ≈ 120 nm). Scale bars = 100 nm. (f) Immunofluorescence assay (IFA) of MDBK cells infected with BCoV‐YBYJ. TRITC fluorescence indicates viral antigen‐positive cells; no signal in mock controls (40×).

**Figure:**
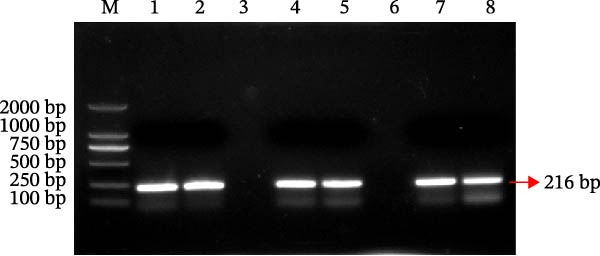
(a)

**Figure:**
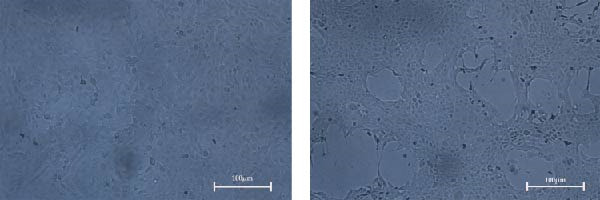
(b)

**Figure:**
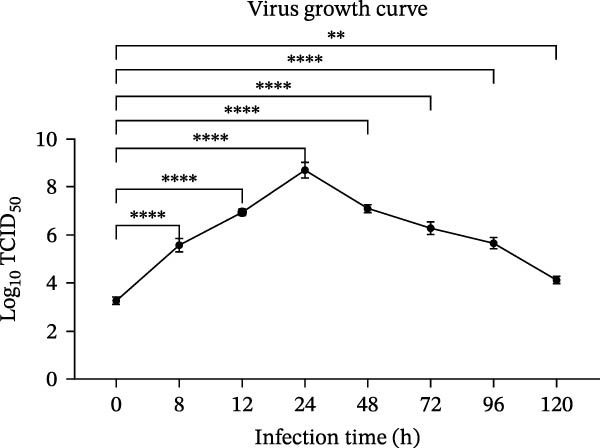
(c)

**Figure:**
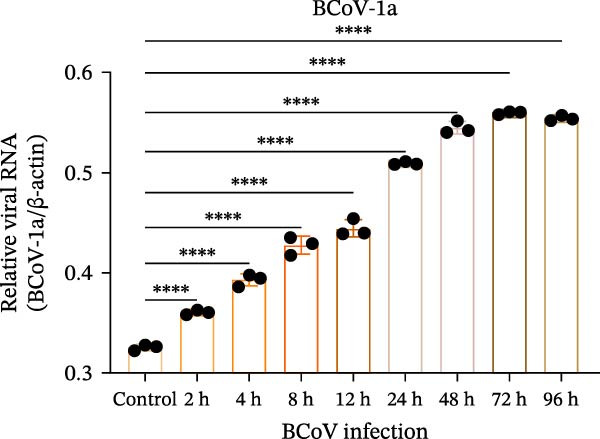
(d)

**Figure:**
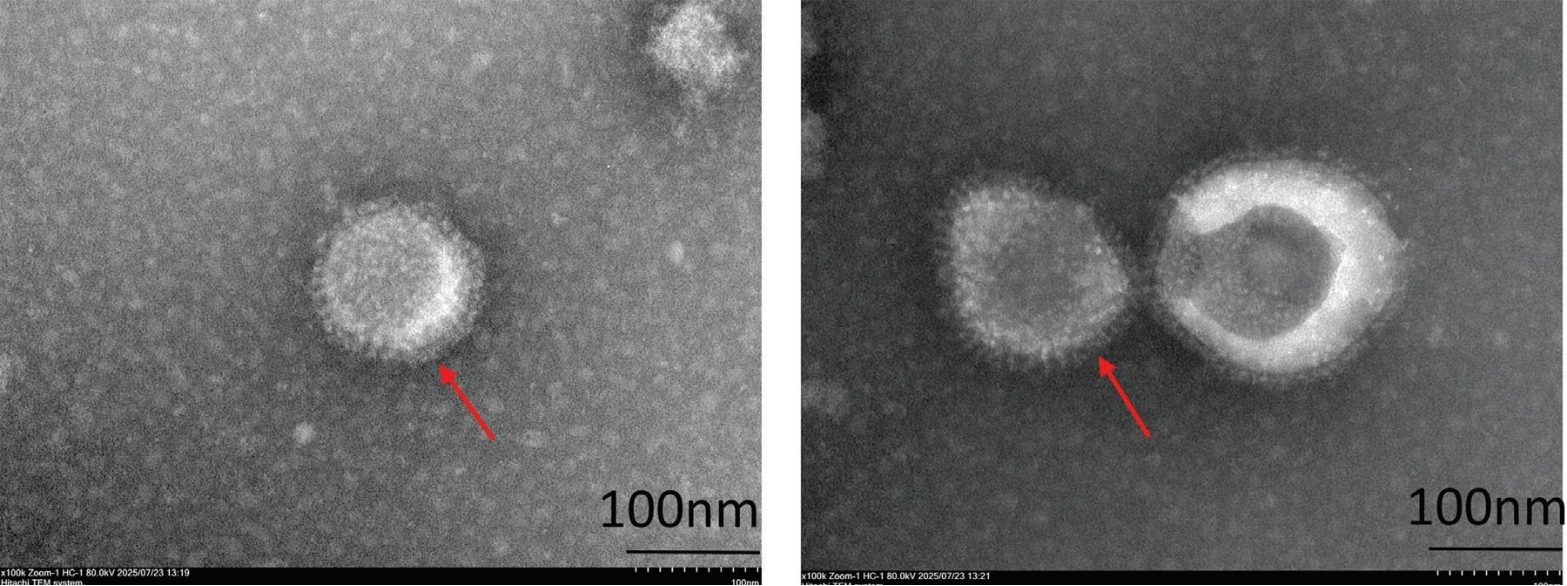
(e)

**Figure:**
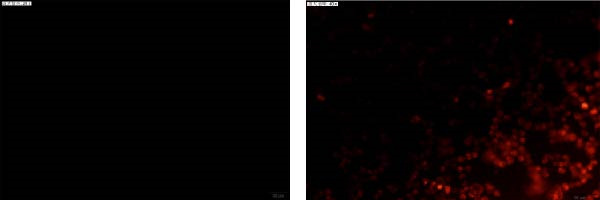
(f)

Following serial passaging in MDBK cells, BCoV‐YBYJ induced typical CPE by 72 h.p.i.: infected cells became rounded, aggregated into clusters, and detached from the culture surface, leading to progressive clearance of the monolayer, whereas uninfected controls showed no CPE under identical conditions (Figure 1b). Viral titration yielded a TCID_50_ of 10^7^ TCID_50_/mL, and single‐step growth analysis (MOI = 5) revealed an early exponential rise in viral titers, peaking at 24 h before gradually stabilizing by 72 h (Figure 1c).

Consistent with the viral growth kinetics, RT‐qPCR quantification of relative ORF1a RNA levels (2^−ΔΔCt^, normalized to β‐actin and calibrated to mock‐infected cells) revealed a marked increase beginning at 4 h.p.i., which continued to rise and reached its peak at 72 h (Figure 1d). These findings indicate progressive accumulation of ORF1a RNA during MDBK cell infection and, together with the rise in infectious titers, support productive replication.

At the 10th passage, viral replication was further validated by TEM and IFA. TEM of the concentrated viral suspension revealed spherical, enveloped, pleomorphic coronavirus‐like particles with characteristic club‐shaped surface projections (spikes). The measurements were predominantly within 62–210 nm, averaging ~120 nm in diameter, which is consistent with previous reports (Figure 1e). In parallel, IFA detected strong fluorescence signals in infected MDBK cells, whereas uninfected controls showed no detectable fluorescence (Figure 1f).

Overall, these results confirm the successful isolation of the BCoV‐YBYJ strain and demonstrate productive replication in MDBK cells.

### 3.2. Phylogenetic Analysis

The geographical location of the sampling sites in Yanji, Jilin Province, China is shown in Figure 2a. Phylogenetic analyses were performed in MEGA using a ML framework with 1000 bootstrap replicates. In the ORF1a‐based tree (216 bp), the Yanbian isolate (BCoV‐ORF1a‐YBYJ) fell within the BCoV ORF1a diversity represented in the reference set and showed closest affinity to East Asian sequences, positioned adjacent to Japanese references (e.g., LC494176.1) and to Chinese references (e.g., OR077310.1; MN982198.1) (Figure 2b). This placement indicates that the ORF1a fragment of BCoV‐YBYJ is most similar to currently available BCoV ORF1a sequences from East Asia in GenBank. Similar regional clustering has been reported in other BCoV molecular epidemiology studies [18].

Figure 2Geographic origin and maximum‐likelihood phylogenetic analyses of BCoV‐YBYJ. (a) Sampling location in Yanji City, Jilin Province, China. (b–d) Maximum‐likelihood phylogenetic trees reconstructed in MEGA with 1000 bootstrap replicates based on partial BCoV sequences obtained in this study: (b) ORF1a fragment (216 bp; GenBank Accession: PX259842.1), (c) S‐gene fragment (1320 bp; GenBank Accession: PZ099269), and (d) M‐gene fragment (672 bp; GenBank Accession: PZ099268). The Yanbian isolate is highlighted in red. In subpart (b), BCoV‐ORF1a‐YBYJ is positioned adjacent to East Asian reference sequences (e.g., Japan LC494176.1 and China OR077310.1/MN982198.1). In subpart (c), the S‐gene fragment (BCoV‐S‐YBYJ3) clusters in close proximity to an Eurasian reference from Kazakhstan (PX450088.1). In subpart (d), the M‐gene fragment (BCoV‐M‐YBYJ) groups most closely with recent Chinese references (OR621177.1 and OR621178.1). Trees are interpreted as evidence of genetic relatedness based on partial fragments and available reference sampling, and not as proof of specific transmission routes.

**Figure:**
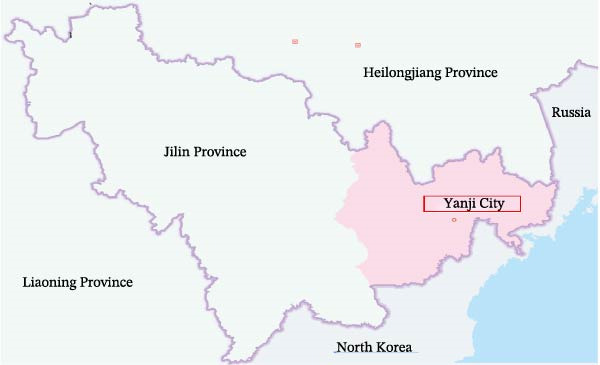
(a)

**Figure:**
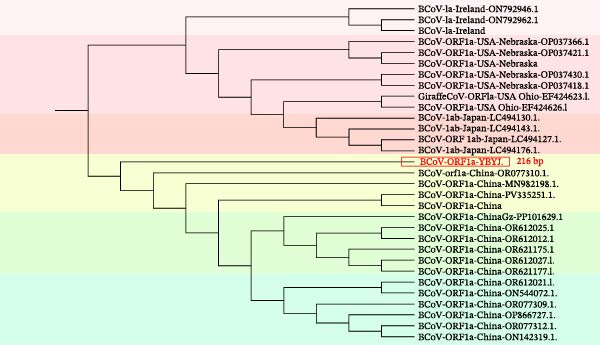
(b)

**Figure:**
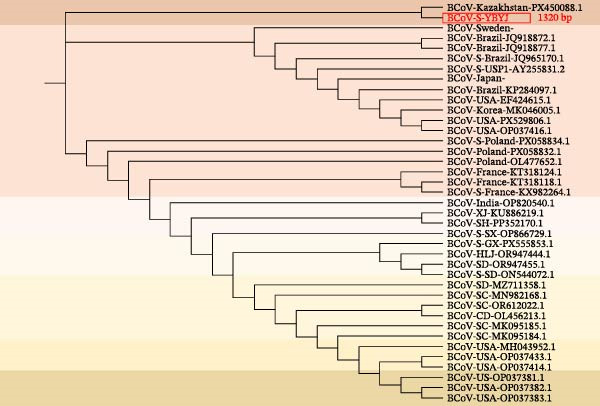
(c)

**Figure:**
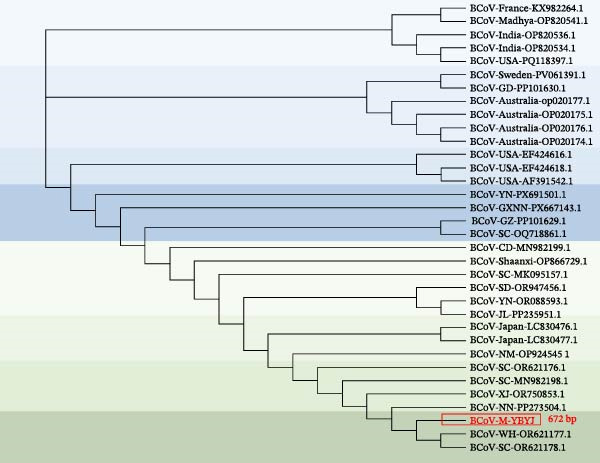
(d)

To increase phylogenetic resolution beyond the short ORF1a fragment, additional loci were sequenced and analyzed. The S‐gene fragment (1320 bp) placed the Yanbian sequence (BCoV‐S‐YBYJ3) within the BCoV S clade and in close proximity to a Eurasian reference sequence from Kazakhstan (PX450088.1) (Figure 2c). Similarly, the M‐gene fragment (672 bp) placed BCoV‐M‐YBYJ within the BCoV M clade and most closely related to two recent Chinese references (OR621177.1 and OR621178.1) (Figure 2d). Across the three loci (ORF1a, S, and M), the concordant clustering within established BCoV clades supports the genetic relatedness of the Yanbian isolate to contemporary BCoV lineages represented in public databases.

Because these phylogenies are based on partial gene fragments and available reference sequences are uneven across regions and time, we interpret the results as evidence of genetic relatedness rather than as proof of specific transmission routes or cross‐border dissemination. Given that NCD is often multifactorial and coinfections are frequently reported, we caution against over‐interpreting single‐locus phylogenies without broader epidemiologic context [19].

At the molecular level, RT‐qPCR targeting ORF1a quantifies viral RNA, whereas IFA demonstrates viral protein expression in infected MDBK cells. Together with growth kinetics and infectious titers, these findings support the replication competence of the isolated BCoV‐YBYJ strain in vitro, consistent with its placement within BCoV lineages inferred from the ORF1a/S/M phylogenies.

### 3.3. Exploratory Tissue Alterations and Viral RNA Detection in BALB/c Mice

To explore tissue alterations following high‐dose oral exposure, BALB/c mice were inoculated with BCoV‐YBYJ and compared with mock controls (*n* = 3/time point). Because BCoV is associated with both enteric and respiratory disease manifestations in cattle [20], we prioritized the colon and lung as representative target organs for exploratory molecular quantification and histopathology in this nonnatural host model. In addition, diarrheal disease is an important contributor to morbidity and reduced performance in calves [21], supporting the emphasis on intestinal readouts. Gross inspection at 7 d.p.i. showed mild colonic congestion/edema and patchy pulmonary changes. Histopathology identified mild‐to‐moderate lesions in the colon and lung, which were summarized using a blinded semiquantitative scoring scheme (Figure 3b). Viral RNA was detected in the colon and lung by RT‐qPCR and is presented as log_10_(2^−ΔCt^) normalized to GAPDH (Figure 3c). In addition, virus recovery was attempted from the colon and lung at 4 and 7 d.p.i. by inoculation of 0.22‐µm‐filtered tissue homogenates onto MDBK cells. CPE was observed, and BCoV RNA was detected in culture supernatants by RT‐qPCR (Supporting Information 6: Table S4, Supporting Information 8: Dataset S4, and Supporting Information 9: Dataset S5), whereas mock‐MDBK controls remained negative.

Figure 3Exploratory colon and lung alterations and viral RNA detection in BALB/c mice following high‐dose oral exposure to BCoV‐YBYJ. BALB/c mice were orally inoculated with 4 × 10^8^ TCID_50_ of BCoV‐YBYJ; DMEM‐treated mice served as controls (*n* = 3 per time point). (a) Representative gross appearance (upper) and H&E‐stained sections (lower) of colon and lung at 4 and 7 d.p.i. Insets show cropped, magnified views of the boxed regions in the 7 d.p.i. panels. Scale bars, 50 µm. (b) Blinded semiquantitative histopathology scores (0–4) for colon and lung at 4 and 7 d.p.i. Five blinded observers independently scored each section. Each dot represents one blinded observer (observer‐level mean score; average of the three criteria) for the indicated group/time point, and bars indicate mean ± SD across observers. Scores are presented descriptively given the limited biological sample size (*n* = 3 mice per group/time point). Histopathology was not scored at 1 d.p.i. (c)Viral RNA levels in colon and lung measured by RT‐qPCR and expressed as log_10_(2^−ΔCt^) (BCoV/GAPDH). Data are shown as individual values with mean ± SD (*n* = 3). Ctrl represents the mock control group processed in parallel (combined as a single baseline control for visualization). Exploratory comparisons across time points were performed within each tissue using one‐way ANOVA followed by Dunnett’s multiple‐comparisons test versus Ctrl; selected comparisons are indicated in the figure. ns, not significant;  ^∗^ *p* < 0.05;  ^∗∗^ *p* < 0.01;  ^∗∗∗^ *p* < 0.001.

**Figure:**
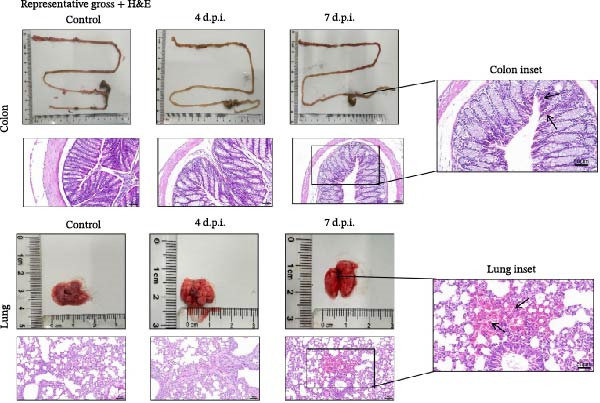
(a)

**Figure:**
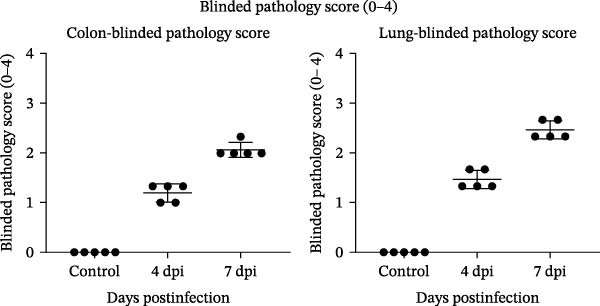
(b)

**Figure:**
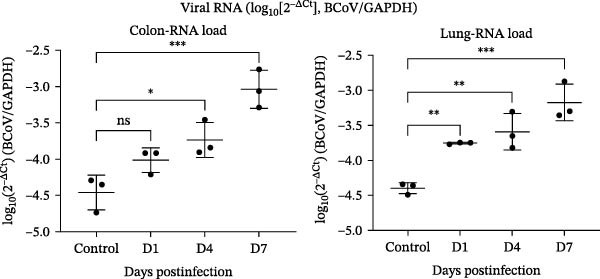
(c)

Together, these findings indicate RT‐qPCR‐detectable signals and recovery‐culture‐associated readouts under the experimental workflow, but they do not establish productive in vivo replication (e.g., residual inoculum, carryover, or low‐level contamination cannot be excluded). Intestinal IHC for viral antigen was negative. This could reflect low antigen abundance, focal distribution, sampling time, or assay sensitivity; therefore, negative IHC does not preclude RT‐qPCR detection of viral RNA. Because mice are not a natural host for BCoV and the inoculation dose was supraphysiologic, these observations are presented as exploratory and are not intended to infer natural‐host tissue tropism or pathogenicity.

Consistent with the blinded scores, colon lesions showed mucosal inflammatory‐cell infiltration with focal epithelial injury and submucosal edema, whereas lung lesions showed interstitial edema with septal thickening and focal inflammatory infiltrates (Figure 3a,b).

In summary, following high‐dose oral exposure in a nonnatural host, BCoV‐YBYJ was associated with detectable viral RNA and mild‐to‐moderate colon and lung lesions; these findings are exploratory and do not establish systemic infection or natural‐host tissue tropism. Raw mouse tissue RT‐qPCR Ct values are provided in Supporting Information 7: Data S2.

### 3.4. Comprehensive Optimization and Validation of RT‐qPCR Assays for Bovine Enteric Viruses

Using correctly sequenced recombinant plasmids as positive controls, conventional RT‐PCR amplification yielded single bright bands of the expected sizes, consistent with established methods [22]. Serial 10‐fold dilutions showed detection limits of 10^5^ copies/µL for BCoV, 10^6^ copies/µL for BRV, and 10^3^ copies/µL for BPV. For assay optimization, the highest plasmid concentrations used were 10^7^ copies/µL (BCoV), 10^9^ copies/µL (BRV), and 10^7^ copies/µL (BPV). Annealing temperature optimization identified 61.1°C for BCoV, 58.7°C for BRV, and 61.1°C for BPV as the most suitable conditions, while primer concentrations of 1.0 µM for BCoV, 0.9 µM for BRV, and 1.0 µM for BPV were found to be optimal.

Standard curves constructed within the primary laboratory run (10^1^–10^7^ copies/µL) showed excellent linearity and acceptable amplification efficiencies (Figure 4a) [23]. Sensitivity testing confirmed a minimum detection threshold of 10^1^ copies/µL (Figure 4b). Specificity within the assay design was supported by expected melt‐curve profiles and the absence of amplification in no‐template controls (Figure 4c). Reproducibility was assessed by intra‐/inter‐assay CV across different days and operators (Table 2). Interlaboratory repeatability was explored using the same plasmid dilution series (see Supporting Information 4: Results S3). Because baseline/threshold settings are instrument‐ and software‐dependent, cross‐site results are interpreted primarily as Ct‐copy‐number concordance and linearity rather than definitive efficiency estimates.

Figure 4Analytical performance of SYBR Green I‐based RT‐qPCR assays for BCoV, BRV, and BPV. Ct values from field diarrheic calf specimens are shown descriptively (no independent reference method applied). (a) Standard curves generated using 10‐fold serial dilutions of plasmid standards (10^7^–10^1^ copies/µL) for each target. (b) Analytical sensitivity shown by amplification curves across the dilution series. (c) Representative amplification/melt‐curve profiles for positive controls and no‐template controls (NTC), supporting assay specificity within the primer designs. (d) Ct distributions for BCoV RT‐qPCR in field specimens from diarrheic calves (*n* = 200), stratified by specimen type (nasal vs. anorectal swabs). Each dot represents one specimen; dashed line indicates the positivity cutoff (Ct = 35). Results are presented descriptively; no independent reference method or systematic coinfection testing was applied to these specimens.

**Figure:**
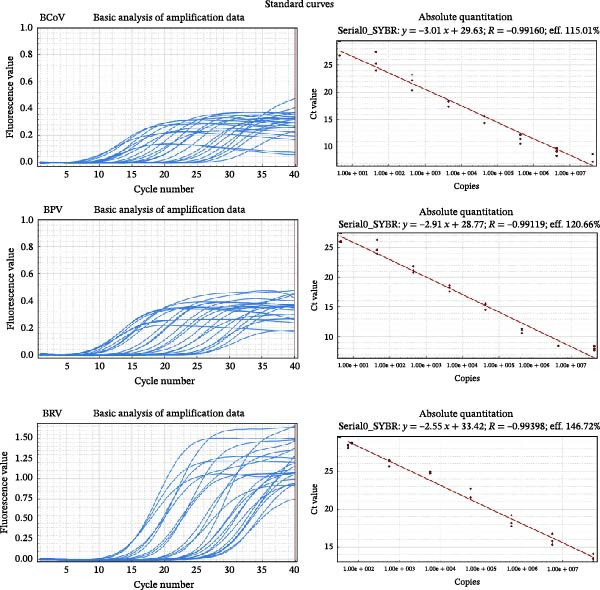
(a)

**Figure:**
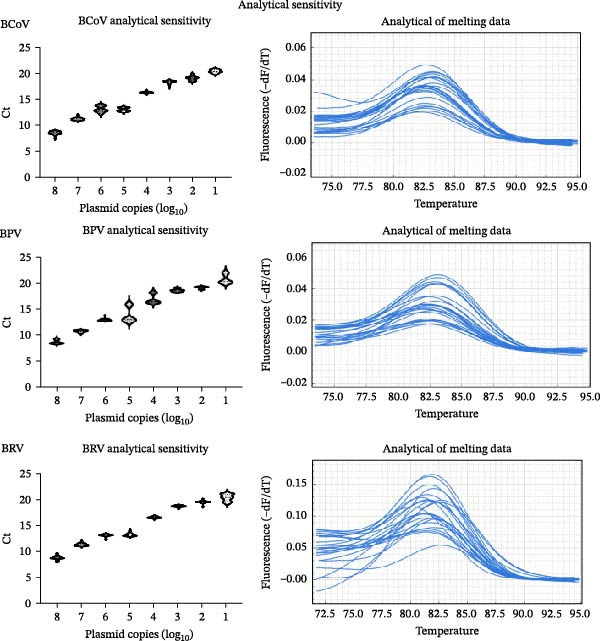
(b)

**Figure:**
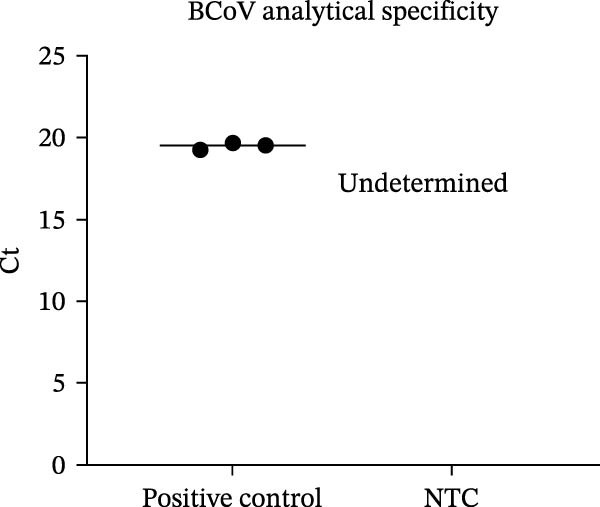
(c)

**Figure:**
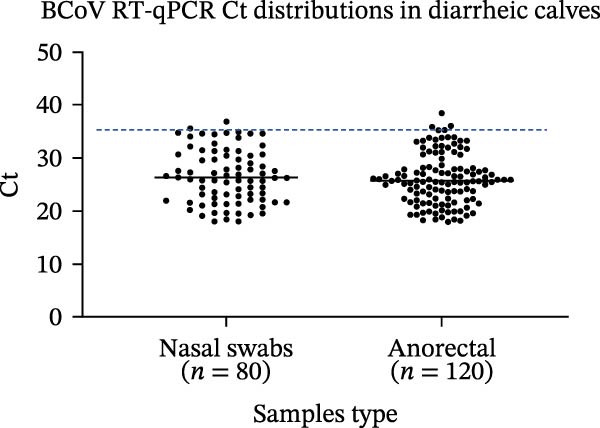
(d)

**Table 2:** Intra‐ and inter‐assay reproducibility of the SYBR Green I‐based RT‐qPCR assays.

Name	Number of standard templates (copies/µL)	Intra‐assay			SD	CV (%)	Inter‐assay			SD	CV (%)
		1	2	3			1	2	3		
BCoV	5.64 × 10^7^	13.54	12.24	11.09	12.29 ± 1.22	9.96	12.23	12.21	12.28	12.24 ± 0.04	0.32
	5.64 × 10^6^	15.94	14.58	14.46	14.99 ± 0.82	5.48	14.55	14.58	14.36	14.50 ± 0.12	0.83
	5.64 × 10^5^	18.38	17.90	17.36	17.88 ± 0.51	2.86	18.11	17.84	17.74	17.90 ± 0.19	1.05
BPV	4.45 × 10^7^	6.39	7.95	6.52	6.95 ± 0.87	12.49	6.37	6.41	6.39	6.39 ± 0.02	0.31
	4.45 × 10^6^	9.52	10.63	10.08	10.08 ± 0.56	5.51	9.54	9.56	9.38	9.52 ± 0.10	1.01
	4.45 × 10^5^	13.19	12.77	13.56	13.17 ± 0.40	3.02	13.06	13.24	13.26	13.19 ± 0.11	0.85
BRV	4.41 × 10^7^	8.05	8.22	10.49	8.92 ± 1.36	15.26	8.69	8.70	7.28	8.22 ± 0.82	9.98
	4.41 × 10^6^	8.53	9.13	9.33	9.00 ± 0.42	4.62	9.81	9.24	8.36	9.13 ± 0.73	8.03
	4.41 × 10^5^	11.06	11.44	14.40	12.30 ± 1.83	14.85	12.25	11.51	10.58	11.44 ± 0.83	7.25

Field sample screening using the optimized BCoV RT‐qPCR. Nasal and anorectal swabs collected from diarrheic calves (200 clinical specimens; nasal swabs *n* = 80; anorectal swabs *n* = 120) were tested with the optimized BCoV RT‐qPCR to describe Ct distributions by specimen type (Figure 4d). BCoV RNA was detected in 70% of diarrheic calf specimens; this high positivity rate likely reflects targeted sampling during suspected outbreaks and should not be extrapolated to population prevalence or interpreted as animal‐level prevalence, because specimens were targeted submissions and were not necessarily paired per animal. An independent reference method and systematic coinfection testing were not applied to these specimens; therefore, the results are presented descriptively rather than as a clinical performance evaluation of the assay.

## 4. Discussion

NCD is an economically important syndrome in cattle production, and BCoV is frequently detected among implicated pathogens. However, locally tailored countermeasures (including vaccines) and region‐specific molecular resources remain limited. Yanbian, which hosts large‐scale production of the regionally important Yanbian yellow cattle and sits at the Sino–Korean–Russian border, highlights the value of actionable diagnostics and strain‐resolved surveillance. Here, we characterize a newly isolated BCoV strain (BCoV‐YBYJ) from Yanbian and develop SYBR Green I‐based RT‐qPCR assays for differential detection of BCoV, BRV [24], and BPV [25]. To our knowledge, this is the first report of a BCoV isolate from Yanbian, addressing an underrepresented area in available sequence datasets and highlighting the importance of regional molecular surveillance [26]. The availability of a local working stock enabled controlled investigations of replication kinetics and cytopathology in MDBK cells, supported by orthogonal confirmation via TEM and IFA, and a proof‐of‐concept in vivo assessment in BALB/c mice to descriptively examine colon and lung findings under high‐dose exposure.

From a virological standpoint, BCoV‐YBYJ displayed robust replication in MDBK cells, with typical CPEs appearing by 72 h.p.i. a single‐step growth peak at 24 h.p.i. and a sustained plateau thereafter. RT‐qPCR targeting ORF1a RNA tracked infectious titers, indicating progressive accumulation of viral genomes and productive replication during early‐to‐mid infection. These kinetic features are broadly consistent with prior descriptions of betacoronavirus growth in bovine cells, and they provide practical benchmarks for in vitro challenge experiments and antiviral screening using a strain that is genetically representative of the local ecology. Importantly, verification by TEM (enveloped, pleomorphic particles with club‐shaped projections) and specific IFA signals in infected monolayers support that the observed CPE and molecular readouts derive from bonafide BCoV infection rather than culture artifacts or contaminants.

In vivo, the BALB/c mouse experiment was designed as an exploratory assessment under high‐dose oral exposure and should not be interpreted as a natural‐host pathogenicity model. Mouse studies are sometimes used as pragmatic, controlled exposure systems for initial in vivo screening, including acute exposure/toxicity testing frameworks that help contextualize tissue‐level readouts under defined experimental conditions [27]. Imaging‐based approaches in mouse systems can further support visualization of infection‐associated signals in vivo under standardized settings [28]. In addition, BALB/c mice have been used to evaluate intestinal phenotypic responses following high‐dose oral exposures, providing methodological context for interpreting gastrointestinal readouts in a standardized mouse setting [29]. Interpretation of organ‐level measurements also benefits from considering baseline variability reported in standardized mouse studies [30]. Accordingly, we present the quantified, blinded histopathology scores and normalized viral RNA readouts (Figure 3) descriptively to document colon and lung findings under these controlled conditions. Confirmation in the natural host (e.g., calves), together with antigen localization and quantification of infectious virus (including serial passaging), will be required to establish cattle‐relevant tissue tropism and disease significance.

The diagnostic component of our study addresses a practical challenge in NCD [31]: overlapping clinical syndromes caused by cocirculating viruses. The three single‐plex SYBR Green I‐based RT‐qPCR assays were designed against conserved regions (BCoV‐ORF1a, BRV‐VP6, and BPV‐NS1), allowing rapid [32], laboratory‐friendly implementation without probe procurement. The analytical performance was strong within the validated dynamic range, and the repeatability metrics were within accepted limits, with the expected increase in variability at low copy numbers, particularly for BRV near the detection limit. The BCoV assay detected BCoV RNA in 70% of targeted diarrheic calf submissions [33], which is reported descriptively and should not be interpreted as population prevalence or etiologic attribution.

Phylogenetic analyses based on ORF1a, S (1320 bp), and M (672 bp) fragments consistently placed BCoV‐YBYJ among contemporary Chinese lineages, supporting regional genetic relatedness. Because geographic sampling remains sparse—particularly across international borders—our data do not allow inference of specific transmission routes, and we therefore avoid attributing the Yanbian strain to cross‐border transmission based on the current dataset.

This work has limitations. First, the BALB/c mouse experiment used a nonnatural host and a supraphysiologic oral dose; despite recovery attempts and molecular detection, these findings should be considered exploratory and not directly extrapolated to cattle. Second, matrix inhibition and extraction efficiency were not systematically quantified across specimen types, and an exogenous extraction control was not incorporated; future studies should include spiked recovery experiments and internal controls. Third, BRV and BPV assays were analytically validated only and require clinical validation against confirmed positive/negative samples and reference methods before routine diagnostic application. Finally, broader geographic sampling and additional genomic data (ideally whole genomes) will be needed to refine phylogeographic inferences.

In this study, we isolated a BCoV strain from diarrheic calves in Yanbian and characterized its replication in MDBK cells by CPE, growth kinetics, TEM morphology, and IFA. We further developed and validated a SYBR Green RT‐qPCR panel for BCoV, BRV, and BPV using plasmid standards and explored interlaboratory repeatability using the same standard curve set. Because Ct calling (baseline/threshold settings) is instrument‐ and software‐dependent, the cross‐site data are interpreted primarily in terms of linearity and Ct‐copy number concordance rather than as definitive efficiency estimates. Consistent with prior reports supporting the analytical utility of SYBR Green RT‐qPCR approaches, our results suggest that this panel is a practical candidate for differential screening of major viral agents associated with calf diarrhea in settings with limited laboratory infrastructure, while recognizing that broader multisite clinical validation and cross‐platform standardization will be needed to fully establish diagnostic performance [34, 35]. In BALB/c mice, high‐dose oral exposure was associated with detectable viral RNA and mild‐to‐moderate lesions in the colon and lung; however, given the nonnatural host and the absence of antigen detection by IHC, these findings should be interpreted as exploratory and do not establish productive systemic infection. Finally, Ct distributions from field submissions of diarrheic calves are reported descriptively and should not be interpreted as population prevalence or clinical diagnostic performance. Future work should prioritize broader geographic sampling, coinfection profiling, and additional genomic data to better resolve local genetic diversity and epidemiology.

## 5. Conclusion

In summary, we report the first isolation of a BCoV strain from Yanbian, China. BCoV‐YBYJ replicated efficiently in MDBK cells, and ML phylogenies based on partial ORF1a, S, and M loci support regional genetic relatedness within available references. We further established and analytically validated SYBR Green I RT‐qPCR assays for BCoV, BRV, and BPV to support research surveillance and differential screening of calf diarrhea viruses. The BRV and BPV assays underwent analytical validation only and require further clinical evaluation before routine diagnostic use.

## Author Contributions

Conceptualization: Siqi Zhang, Haoyuan Ma, Rui Du, and Xu Gao. Methodology: Siqi Zhang, Haoyuan Ma, Jiawei Zhao, and Kai Yu. Investigation: Siqi Zhang, Haoyuan Ma, Jiawei Zhao, Kai Yu, Jingrui Hao, Hao Yu, Jianyou Jin, Shuoning Cao, Xinpeng Ji, Shujiang Xue, Qiang Li, Zhiqiang Xu, Shengwei Ji, Chenghui Li, Zheng Sun, and Jialiang Xie. Formal analysis: Siqi Zhang, Haoyuan Ma, and Jiawei Zhao. Resources: Rui Du and Xu Gao. Data curation: Jiawei Zhao and Kai Yu. Writing – original draft: Siqi Zhang and Haoyuan Ma. Writing – review and editing: Rui Du and Xu Gao. Visualization: Siqi Zhang and Haoyuan Ma. Supervision: Rui Du and Xu Gao. Funding acquisition: Rui Du and Xu Gao.

## Funding

This work was supported by the National Key Research and Development Program of China (Grant 2023YFD1300103), the Science and Technology Development Project of Jilin Province (Grant YDZJ202203CGZH050), and the Scientific Research Project of the Education Department of Jilin Province (Grant JJKH20250419KJ).

## Ethics Statement

All animal experiments were conducted in accordance with the guidelines of the Institutional Animal Care and Use Committee (IACUC) of Yanbian University. The experimental protocol was reviewed and approved by the Animal Ethics Committee of Yanbian University (Approval Number YD20250827010).

## Conflicts of Interest

The authors declare no conflicts of interest.

## Supporting Information

Additional supporting information can be found online in the Supporting Information section.

## Supporting information


**Supporting Information 1** Table S1: Primers and in silico validation: primer sequences used for BCoV/BRV/BPV RT‐PCR/RT‐qPCR and summary of in silico coverage checks.

**Supporting Information 2** Table S2: S and M sequencing primers: primer sets used to amplify and Sanger sequence partial S and M gene fragments.

**Supporting Information 3** Methods S1: Blinded semiquantitative histopathology scoring: scoring rubric (0–4), criteria definitions, and raw blinded scores.

**Supporting Information 4** Results S3: Interlaboratory repeatability: summary of cross‐laboratory standard curve concordance and interpretation notes.

**Supporting Information 5** Data S3: Interlaboratory standard curves: raw Ct values and standard curve calculations from the independent laboratory.

**Supporting Information 6** Table S4: Reisolation: tissue homogenate inoculation results, CPE observations, and RT‐qPCR confirmation from culture supernatants.

**Supporting Information 7** Data S2: Mouse qPCR raw Ct: raw Ct values for mouse colon/lung RT‐qPCR (BCoV and GAPDH).

**Supporting Information 8** Dataset S4: Reisolation qPCR (ORF1a): raw Ct values for ORF1a RT‐qPCR from culture supernatants during reisolation attempts.

**Supporting Information 9** Dataset S5: Reisolation qPCR (GAPDH): raw Ct values for GAPDH RT‐qPCR from culture supernatants during reisolation attempts.

## Data Availability

The ORF1a, S (1320 bp), and M (672 bp) sequences of BCoV‐YBYJ have been deposited in GenBank under accession numbers PX259842.1, PZ099269, and PZ099268, respectively. Mouse tissue RT‐qPCR raw Ct values are provided as Supporting Information 7: Data S2, and interlaboratory standard‐curve raw data are provided as Supporting Information 5: Data S3, and reisolation culture supernatant RT‐qPCR raw Ct datasets are provided as Supporting Information 8: Dataset S4 (ORF1a) and Supporting Information 9: Dataset S5 (GAPDH). Primer sequences and in silico validation are provided in Supporting Information 1: Table S1, and S/M sequencing primers are provided in Supporting Information 2: Table S2. Blinded pathology scoring criteria (and raw scoring sheets) are provided as Supporting Information 3: Methods S1. Additional data supporting the findings of this study are available from the corresponding authors upon reasonable request.
