# Supplementary material for: First Isolation of Bovine Coronavirus From Yanbian, China, and Analytical Validation of a SYBR Green I RT‐qPCR Panel for Calf Diarrhea Viruses
Source: Transbound Emerg Dis. 2026 Apr 27;2026:6648536. doi: 10.1155/tbed/6648536 (PMC13121856; doi:10.1155/tbed/6648536)
Supplement: Supplementary file 1 — Supporting Information 1 Table S1: Primers and in silico validation: primer sequences used for BCoV/BRV/BPV RT‐PCR/RT‐qPCR and summary of in silico coverage checks. [file TBED-2026-6648536-s008.docx]

**Supplementary Table S1. Primers used in this study and in silico validation summary.**

| Target | Gene/region | Primer | Sequence (5'–3') | Amplicon size (bp) |
| --- | --- | --- | --- | --- |
| BCoV | ORF1a | Forward | AGGAACACCTATTGCCAATTGT | 216 |
| BCoV | ORF1a | Reverse | GGATCTTTTATACCTACAGGCACTTG | 216 |
| BPV | NS1 | Forward | CCAATCGTCCTCTACTGCTT | 110 |
| BPV | NS1 | Reverse | GTGCTCGGTGAGCGCTAAAT | 110 |
| BRV | VP6 | Forward | AGAAGACAAAGAACGGGTTT | 113 |
| BRV | VP6 | Reverse | CACATCGTACCCATCAAGTTAT | 113 |

Primers were designed against conserved regions and evaluated in silico (BLASTn against GenBank; primer–dimer and secondary-structure checks in Primer Premier where applicable). SYBR Green assays were considered specific when a single melt-curve peak at the expected Tm and a sigmoidal amplification curve were observed.

Note: Amplicon sizes were 216 bp for BCoV (ORF1a), 110 bp for BPV (NS1), and 113 bp for BRV (VP6), as used for the SYBR Green RT-qPCR assays in this study.
