# Supplementary material for: First Isolation of Bovine Coronavirus From Yanbian, China, and Analytical Validation of a SYBR Green I RT‐qPCR Panel for Calf Diarrhea Viruses
Source: Transbound Emerg Dis. 2026 Apr 27;2026:6648536. doi: 10.1155/tbed/6648536 (PMC13121856; doi:10.1155/tbed/6648536)
Supplement: Supplementary file 2 — Supporting Information 2 Table S2: S and M sequencing primers: primer sets used to amplify and Sanger sequence partial S and M gene fragments. [file TBED-2026-6648536-s001.docx]

**Supplementary Table S2. Primers used for amplification and sequencing of S and M gene fragments.**

Primers listed below were used for RT-PCR amplification of the BCoV S and M gene fragments, followed by bidirectional Sanger sequencing.

| Target | Gene | Primer | Sequence (5’–3’) | Designed amplicon (bp) | Purpose |
| --- | --- | --- | --- | --- | --- |
| BCoV | S | S-F | TTTTGATACTTTTAATTTCCTTACCAACGACTTTTG | 1316 | RT-PCR + Sanger sequencing |
| BCoV | S | S-R | AACAGAAACATTAGCAGCAGGTAAATTATAATAC | 1316 | RT-PCR + Sanger sequencing |
| BCoV | M | M-F | ATGAGTAGTGTAACCACACCAGCAC | 693 | RT-PCR + Sanger sequencing |
| BCoV | M | M-R | TTAGATATTATTTCTCAACAATGCGGT | 693 | RT-PCR + Sanger sequencing |

Note: After trimming primer sequences and low-quality ends, consensus sequences of 1,320 bp (S) and 672 bp (M) were used for phylogenetic analysis and were submitted to GenBank (NCBI) (submission IDs 3058689 [S] and 3058684 [M]); accession numbers will be updated once assigned.
