# Supplementary material for: First Isolation of Bovine Coronavirus From Yanbian, China, and Analytical Validation of a SYBR Green I RT‐qPCR Panel for Calf Diarrhea Viruses
Source: Transbound Emerg Dis. 2026 Apr 27;2026:6648536. doi: 10.1155/tbed/6648536 (PMC13121856; doi:10.1155/tbed/6648536)
Supplement: Supplementary file 3 — Supporting Information 3 Methods S1: Blinded semiquantitative histopathology scoring: scoring rubric (0–4), criteria definitions, and raw blinded scores. [file TBED-2026-6648536-s002.docx]

**Supplementary Methods S1. Blinded semiquantitative histopathology scoring (0-4).**

Lesions were scored independently by blinded observers using a 0-4 semiquantitative scale applied to each criterion as follows: 0, no lesion; 1, minimal (focal); 2, mild (limited extent); 3, moderate (multifocal to diffuse); 4, severe (widespread tissue damage/architectural disruption).

Scoring criteria (each 0-4):

• Criterion 1: Tissue architecture/structural injury.

• Criterion 2: Inflammatory cell infiltration.

• Criterion 3: Edema and/or tissue structural alteration.

For each tissue section, an overall mean score was calculated as the average of the three criteria. This worksheet reports observer-level mean scores for each organ and time point. Where multiple observers scored the same section(s), results are summarized descriptively as mean ± SD of observer-level mean scores to illustrate inter-observer consistency; observers were treated as measurement repeats and not as independent biological replicates. For Figure 3b visualization, each dot represents one blinded observer.

| **Sample ID** | **Group** | **Organ** | **Criterion1/Structure** | **Criterion2/Inflammation** | **Criterion3/Edema or Architecture** | **Mean Score** | **Observer** |
| --- | --- | --- | --- | --- | --- | --- | --- |
| M1 | Control | Colon | 0 | 0 | 0 | 0 | Z.Q |
| M2 | 4dpi | Colon | 1 | 2 | 1 | 1.333333333 | Z.Q |
| M3 | 7dpi | Colon | 2 | 2 | 2 | 2 | Z.Q |
| M4 | Control | Lung | 0 | 0 | 0 | 0 | Z.Q |
| M5 | 4dpi | Lung | 1 | 2 | 1 | 1.333333333 | Z.Q |
| M6 | 7dpi | Lung | 2 | 3 | 2 | 2.333333333 | Z.Q |
| M7 | Control | Liver | 0 | 0 | 0 | 0 | Z.Q |
| M8 | 4dpi | Liver | 1 | 0 | 1 | 0.666666667 | Z.Q |
| M9 | 7dpi | Liver | 1 | 1 | 1 | 1 | Z.Q |
| M10 | Control | Kidney | 0 | 0 | 0 | 0 | Z.Q |
| M11 | 4dpi | Kidney | 1 | 1 | 0 | 0.666666667 | Z.Q |
| M12 | 7dpi | Kidney | 1 | 1 | 1 | 1 | Z.Q |
| M13 | Control | Spleen | 0 | 0 | 0 | 0 | Z.Q |
| M14 | 4dpi | Spleen | 1 | 1 | 1 | 1 | Z.Q |
| M15 | 7dpi | Spleen | 2 | 2 | 1 | 1.666666667 | Z.Q |
| M1 | Control | Colon | 0 | 0 | 0 | 0 | M.H |
| M2 | 4dpi | Colon | 1 | 1 | 1 | 1 | M.H |
| M3 | 7dpi | Colon | 2 | 2 | 2 | 2 | M.H |
| M4 | Control | Lung | 0 | 0 | 0 | 0 | M.H |
| M5 | 4dpi | Lung | 2 | 2 | 1 | 1.666666667 | M.H |
| M6 | 7dpi | Lung | 2 | 3 | 3 | 2.666666667 | M.H |
| M7 | Control | Liver | 0 | 0 | 0 | 0 | M.H |
| M8 | 4dpi | Liver | 1 | 0 | 1 | 0.666666667 | M.H |
| M9 | 7dpi | Liver | 1 | 1 | 1 | 1 | M.H |
| M10 | Control | Kidney | 0 | 0 | 0 | 0 | M.H |
| M11 | 4dpi | Kidney | 1 | 1 | 1 | 1 | M.H |
| M12 | 7dpi | Kidney | 1 | 1 | 1 | 1 | M.H |
| M13 | Control | Spleen | 0 | 0 | 0 | 0 | M.H |
| M14 | 4dpi | Spleen | 1 | 2 | 1 | 1.333333333 | M.H |
| M15 | 7dpi | Spleen | 2 | 2 | 1 | 1.666666667 | M.H |
| M1 | Control | Colon | 0 | 0 | 0 | 0 | Z.J |
| M2 | 4dpi | Colon | 1 | 2 | 1 | 1.333333333 | Z.J |
| M3 | 7dpi | Colon | 2 | 2 | 3 | 2.333333333 | Z.J |
| M4 | Control | Lung | 0 | 0 | 0 | 0 | Z.J |
| M5 | 4dpi | Lung | 1 | 2 | 1 | 1.333333333 | Z.J |
| M6 | 7dpi | Lung | 2 | 3 | 2 | 2.333333333 | Z.J |
| M7 | Control | Liver | 0 | 0 | 0 | 0 | Z.J |
| M8 | 4dpi | Liver | 1 | 0 | 1 | 0.666666667 | Z.J |
| M9 | 7dpi | Liver | 1 | 1 | 1 | 1 | Z.J |
| M10 | Control | Kidney | 0 | 0 | 0 | 0 | Z.J |
| M11 | 4dpi | Kidney | 1 | 1 | 0 | 0.666666667 | Z.J |
| M12 | 7dpi | Kidney | 1 | 1 | 1 | 1 | Z.J |
| M13 | Control | Spleen | 0 | 0 | 0 | 0 | Z.J |
| M14 | 4dpi | Spleen | 1 | 1 | 1 | 1 | Z.J |
| M15 | 7dpi | Spleen | 2 | 2 | 1 | 1.666666667 | Z.J |
| M1 | Control | Colon | 0 | 0 | 0 | 0 | Y.K |
| M2 | 4dpi | Colon | 1 | 1 | 1 | 1 | Y.K |
| M3 | 7dpi | Colon | 2 | 2 | 2 | 2 | Y.K |
| M4 | Control | Lung | 0 | 0 | 0 | 0 | Y.K |
| M5 | 4dpi | Lung | 2 | 2 | 1 | 1.666666667 | Y.K |
| M6 | 7dpi | Lung | 2 | 3 | 3 | 2.666666667 | Y.K |
| M7 | Control | Liver | 0 | 0 | 0 | 0 | Y.K |
| M8 | 4dpi | Liver | 1 | 0 | 1 | 0.666666667 | Y.K |
| M9 | 7dpi | Liver | 1 | 1 | 1 | 1 | Y.K |
| M10 | Control | Kidney | 0 | 0 | 0 | 0 | Y.K |
| M11 | 4dpi | Kidney | 1 | 1 | 1 | 1 | Y.K |
| M12 | 7dpi | Kidney | 1 | 1 | 1 | 1 | Y.K |
| M13 | Control | Spleen | 0 | 0 | 0 | 0 | Y.K |
| M14 | 4dpi | Spleen | 1 | 2 | 1 | 1.333333333 | Y.K |
| M15 | 7dpi | Spleen | 2 | 2 | 1 | 1.666666667 | Y.K |
| M1 | Control | Colon | 0 | 0 | 0 | 0 | G.X |
| M2 | 4dpi | Colon | 1 | 2 | 1 | 1.333333333 | G.X |
| M3 | 7dpi | Colon | 2 | 2 | 2 | 2 | G.X |
| M4 | Control | Lung | 0 | 0 | 0 | 0 | G.X |
| M5 | 4dpi | Lung | 1 | 2 | 1 | 1.333333333 | G.X |
| M6 | 7dpi | Lung | 2 | 3 | 2 | 2.333333333 | G.X |
| M7 | Control | Liver | 0 | 0 | 0 | 0 | G.X |
| M8 | 4dpi | Liver | 1 | 0 | 1 | 0.666666667 | G.X |
| M9 | 7dpi | Liver | 1 | 1 | 1 | 1 | G.X |
| M10 | Control | Kidney | 0 | 0 | 0 | 0 | G.X |
| M11 | 4dpi | Kidney | 1 | 1 | 0 | 0.666666667 | G.X |
| M12 | 7dpi | Kidney | 1 | 1 | 1 | 1 | G.X |
| M13 | Control | Spleen | 0 | 0 | 0 | 0 | G.X |
| M14 | 4dpi | Spleen | 1 | 1 | 1 | 1 | G.X |
| M15 | 7dpi | Spleen | 2 | 2 | 1 | 1.666666667 | G.X |
