# Supplementary material for: First Isolation of Bovine Coronavirus From Yanbian, China, and Analytical Validation of a SYBR Green I RT‐qPCR Panel for Calf Diarrhea Viruses
Source: Transbound Emerg Dis. 2026 Apr 27;2026:6648536. doi: 10.1155/tbed/6648536 (PMC13121856; doi:10.1155/tbed/6648536)
Supplement: Supplementary file 4 — Supporting Information 4 Results S3: Interlaboratory repeatability: summary of cross‐laboratory standard curve concordance and interpretation notes. [file TBED-2026-6648536-s009.docx]

**Supplementary Results S3. Inter-laboratory repeatability of plasmid standard curves.**

Inter-laboratory repeatability.

To evaluate repeatability across laboratories, standard curves for the SYBR Green I RT-qPCR assays were generated using the same plasmid dilution series. Because baseline correction and thresholding (Ct calling) are platform- and software-dependent, slope-derived efficiency estimates and regression coefficients are not directly comparable across laboratories. We therefore summarize inter-laboratory performance by linearity (R²) and the expected monotonic Ct separation across ten-fold dilutions. Full dilution-series data are provided in Supplementary Data S3.

| Assay | R² | Comment |
| --- | --- | --- |
| BCoV | 0.977 | Ct vs log10(copies) fit |
| BPV | 0.982 | Ct vs log10(copies) fit |
| BRV | 0.982 | Ct vs log10(copies) fit |

Note: Efficiency (%) is not reported for the inter-laboratory comparison because it is not directly comparable across platforms due to instrument/software-specific Ct calling rules; inter-laboratory results are interpreted primarily in terms of R² and Ct concordance across the dilution series.
