# Supplementary material for: First Isolation of Bovine Coronavirus From Yanbian, China, and Analytical Validation of a SYBR Green I RT‐qPCR Panel for Calf Diarrhea Viruses
Source: Transbound Emerg Dis. 2026 Apr 27;2026:6648536. doi: 10.1155/tbed/6648536 (PMC13121856; doi:10.1155/tbed/6648536)
Supplement: Supplementary file 6 — Supporting Information 6 Table S4: Reisolation: tissue homogenate inoculation results, CPE observations, and RT‐qPCR confirmation from culture supernatants. [file TBED-2026-6648536-s005.docx]

**Supplementary Results: Virus re-isolation and RT-qPCR confirmation (mouse tissues → MDBK)**

To provide evidence beyond tissue RNA detection, selected tissue homogenates from infected and mock-treated mice were inoculated onto MDBK cells. Cell culture supernatants from these re-isolation cultures were subjected to SYBR Green I RT-qPCR targeting BCoV ORF1a. In parallel, GAPDH RT-qPCR was performed as an internal control for RNA presence/RT-qPCR performance in the corresponding preparations. As summarized in Table S4, all mock-derived cultures were ORF1a-undetectable, whereas cultures derived from infected animals (colon and lung at 4 and 7 dpi) were consistently ORF1a-positive. Full raw Ct values are provided in Dataset S4-Reisolation qpcr (ORF1a) and Dataset S5-GAPDH-Reisolation qpcr (GAPDH).

**Supplementary Table S4. Re-isolation culture supernatant RT-qPCR results (ORF1a) with GAPDH control**

*Notes: Ct values are mean ± SD of technical triplicates. “Undet” indicates no amplification within the run.*

| **Sample**  **(Dataset label)** | **Group** | **Tissue** | **dpi** | **ORF1a Ct**  **(mean ± SD)** | **ORF1a Ct range**  **(min–max)** | **GAPDH Ct**  **(mean ± SD)** | **GAPDH Ct range**  **(min–max)** |
| --- | --- | --- | --- | --- | --- | --- | --- |
| Colon-Mock-1 | Control (mock) | Colon | – | Undet | – | 19.24 ± 0.04 | 19.19–19.27 |
| Colon-Mock-2 | Control (mock) | Colon | – | Undet | – | 18.87 ± 0.50 | 18.31–19.25 |
| Colon-Mock-3 | Control (mock) | Colon | – | Undet | – | 19.20 ± 0.45 | 18.87–19.71 |
| Colon-4dpi-1 | Infected | Colon | 4 | 18.78 ± 0.87 | 17.80–19.45 | 17.35 ± 0.16 | 17.24–17.53 |
| Colon-4dpi-2 | Infected | Colon | 4 | 18.43 ± 0.55 | 17.84–18.92 | 18.01 ± 0.49 | 17.47–18.43 |
| Colon-4dpi-3 | Infected | Colon | 4 | 18.61 ± 0.14 | 18.53–18.78 | 18.56 ± 0.11 | 18.43–18.62 |
| Colon-7dpi-1 | Infected | Colon | 7 | 19.87 ± 0.45 | 19.49–20.37 | 18.29 ± 0.30 | 18.09–18.64 |
| Colon-7dpi-2 | Infected | Colon | 7 | 21.74 ± 0.73 | 21.24–22.58 | 17.73 ± 0.57 | 17.08–18.10 |
| Colon-7dpi-3 | Infected | Colon | 7 | 20.83 ± 0.53 | 20.51–21.45 | 18.91 ± 0.38 | 18.47–19.17 |
| Lung-Mock-1 | Control (mock) | Lung | – | Undet | – | 18.26 ± 0.13 | 18.16–18.41 |
| Lung-Mock-2 | Control (mock) | Lung | – | Undet | – | 18.22 ± 0.16 | 18.04–18.35 |
| Lung-Mock-3 | Control (mock) | Lung | – | Undet | – | 18.66 ± 0.36 | 18.26–18.94 |
| Lung-4dpi-1 | Infected | Lung | 4 | 20.20 ± 0.61 | 19.60–20.82 | 18.95 ± 0.41 | 18.53–19.35 |
| Lung-4dpi-2 | Infected | Lung | 4 | 20.67 ± 0.63 | 20.28–21.39 | 18.72 ± 0.24 | 18.46–18.92 |
| Lung-4dpi-3 | Infected | Lung | 4 | 20.30 ± 0.11 | 20.19–20.41 | 19.60 ± 0.27 | 19.39–19.90 |
| Lung-7dpi-1 | Infected | Lung | 7 | 19.66 ± 0.26 | 19.42–19.94 | 18.99 ± 0.30 | 18.81–19.34 |
| Lung-7dpi-2 | Infected | Lung | 7 | 20.28 ± 0.10 | 20.17–20.37 | 16.62 ± 0.06 | 16.58–16.69 |
| Lung-7dpi-3 | Infected | Lung | 7 | 21.85 ± 0.14 | 21.72–22.00 | 19.59 ± 0.26 | 19.29–19.77 |
